# Epidemic of chronic diseases and the related healthy lifestyle interventions in rural areas of Shandong Province, China

**DOI:** 10.1186/s12889-020-08729-y

**Published:** 2020-05-01

**Authors:** Juncheng Lyu, Wen Zhang, Wei Li, Suzhen Wang, Jie Zhang

**Affiliations:** 1 Department of Public Health, Weifang Medical University, Weifang, 261053 Shandong China; 2 Department of Psychiatry, Binzhou People’s Hospital, Binzhou, Shandong China; 3 School of Public Health, Shandong University, Jinan, 250000 China; 4 Department of Sociology, State University of New York College at Buffalo, Buffalo, USA

**Keywords:** Chronic diseases, Healthy lifestyle intervention, Epidemiology, Health promotion, Rural China

## Abstract

**Background:**

There were amounts of previous studies on chronic diseases, but few studies on the prevalence of chronic disease and the healthy lifestyle intervention in recent years, China. This study aimed to investigate the prevalence of chronic disease and the implementation of healthy lifestyle intervention in rural areas of China, so as to put forward health promotion measures to control the chronic diseases effectively.

**Method:**

A large cross-sectional study (*N* = 2168) on community diagnosis and chronic disease was carried out in Shandong province, China. The chronic disease questionnaire and the healthy lifestyle intervention questionnaires were recruited to survey the chronic diseases and the implementation of healthy lifestyle intervention. Physical examination and biochemical indicators examination were carried out by the medical staffs and clinical laboratory.

**Results:**

The current diagnosed prevalence of hypertension, diabetes, hyperlipidemia for total sample, female, male were 24.97, 24.6, 25.5, 7.60, 8.9, 6.0 and 40.27%, 45.9, 33.3% respectively in rural China. The one-year prevalence of myocardial infarction (MI) and stroke of the total sample, female, male were 1.06, 1.0, 1.1 and 2.09%, 2.2, 2.0% respectively.

Healthy lifestyles interventions were not effective in rural China. The current active smoking rate and passive smoking rate were 25.68 and 42.65%. 27.86% of the population drunk alcohol within a month and 47.01% of them participated in the actions to control salt daily intake. Only 1.07 and 7.89% of the population participated in medium to high intensity physical exercises.

**Conclusions:**

The prevalence of common chronic diseases were still high and the implementation of healthy lifestyle intervention were not optimal in rural area, China. Challenges to prevent chronic diseases were still severe, so medical institutes, government and individuals would put forward effective strategies to reduce the prevalence and public health promotion project should be effectively strengthened.

## Background

Chronic diseases, such as hypertension, heart disease, stroke, cancer, diabetes and chronic respiratory diseases, were the leading cause of mortality in the world, accounting for 63% of all deaths [1]. Meanwhile, mortality of middle-aged Chinese people caused by chronic disease was higher than the developed countries [2]. It was estimated that 80% of deaths and 70% of disability-adjusted life-years (DALYs) lost in China came from chronic disease in 2005, and 160 million adults were hypertensive [3]. As we all know, the aging of population in China increase the harm of chronic diseases. The sixth national population census showed that there were 178 million old people (≥60 years old), which was 13.26% of the population in China. The World Bank had projected that more than 400 million Chinese people could be over 60 years old by 2050. With the rapid development of economy, environmental changes [4] and the ageing tendency, unhealthy lifestyle such as tobacco using, unhealthy nutrition, and physical inactivity constantly spring up. Now, China is facing the formidable challenge of chronic diseases.

The National Basic Public Health Service Project is an important part of promoting equalization of health services and deepening reform of the medical and health care system. It is provided and funded by the Chinese government to all the residents free of charge, and focusing on children, pregnant women, the elderly and patients with chronic diseases. At present, the National Basic Public Health Service Project includes 15 basic public health services. Though Chinese government has invested enormous capital resources on the prevention of chronic diseases in recent decades, there is no obvious decrease in the prevalence of common chronic diseases such as hypertension, cardiovascular disease, and stroke.

Previous studies had reported various studies related to chronic diseases in China. Jing, Lou and Zhao etc. assessed the economic burden of chronic hepatitis B (CHB)-related diseases in Beijing and Guangzhou, China [5]. Li, Y., et al. studied the risk factors for non-communicable chronic diseases in women in China [6]. The prevalence and risk factors of chronic obstructive pulmonary diseases in Hainan island of China [7] and risk factors of chronic non-communicable diseases still was studied [8]. Ning, G. and Bloomgarden, Z. reported the prevalence, diagnosis and control of diabetes in China [9]. Hung, K.K. et al., researched the disease pattern and chronic illness in rural China of Sichuan after 2008 earthquake [10]. Tian, M., et al., reported the chronic disease knowledge and its determinants among chronically ill adults in rural areas of Shanxi province in China [11], Kong, L.Z., studied the strategy adjustment and actions of non-communicable chronic diseases in China [12]. The chronic disease prevalence for elders in Beijing was researched by Liu et al. [13]. Gao reported the study on chronic diseases among middle-aged and elderly population in Shanghai [14]. Cheng et al. researched the chronic disease mortality in rural and urban residents in Hubei province [15].

There were still lot of previous literatures on the relationship between chronic disease and the health lifestyles. Population attributable risks of cigarette smoking for deaths of chronic diseases among adults aged 40–74 years in urban Shanghai [16] and prevalence of tobacco smoking and determinants of success in quitting smoking among patients with chronic diseases in rural western China [17] have been published. The breast cancer risks of Chinese women relationship with active, passive smoking and alcohol drinking was researched [18]. The Physical activity and chronic diseases among older people in a mid-size city in China still was reported in previous literature [19]. The public policy implication of preventing chronic diseases by promoting healthy diet and lifestyle had been introduced [20]. The previous literature [21] emphasized the central role of preventive medicine and healthy lifestyle behaviors to combat chronic diseases in Sri Lanka.

Although many previous studies on chronic diseases had been carried, there were few studies on the prevalence of chronic disease and implementation of healthy lifestyle intervention in recent year in rural China. Meanwhile, little previous literatures simultaneously research chronic diseases and healthy lifestyle intervention. This study aimed to survey the prevalence of chronic disease and the implementation of healthy lifestyle intervention, so as to evaluate the preventive effects and bring forward health promotion measures to control the chronic disease in rural China.

## Methods

### Study design, setting and participants

Data of the current study (*N* = 2168) were collected from a large cross-sectional study on community diagnosis for chronic disease in rural areas of Shandong province, China. Shandong province located on the east coast of China, and with economic prosperity both in industry and agriculture. The random stratified cluster sampling method was applied to select the survey subjects. Three counties of Shandong province and three villages in each country were chosen randomly according to the development of economy and the geographical location. Then, all the residents in the nine villages were chosen as the survey respondents.

In order to control the information bias and loss to follow-up, the collaboration institutes research meeting had been held several times to discuss the quality control. Representatives of local sanitary bureau, health agency, village administration and researcher designer were invited to attend the meeting. Pilot investigation was carried out to test the reliability and validity of the questionnaires. The interviewers were trained in order to increase the skill of interviewer. The medical professional experts, university teachers and the postgraduate students majoring in preventive medicine were recruited to carry out the face-to-face questionnaire interview. The village health agencies staffs were responsible for physical examination. The biochemical blood samples were drawn by the local county hospital medical staffs. The biological samples were transported quickly to the local county hospital and analyzed by biochemical equipment.

### Questionnaire measurements

The chronic disease questionnaire, designed by Chinese Center for Disease Control and Prevention (China CDC) was recruited to survey the items about chronic diseases. The questionnaire consists of family information part and individual information part. The family interviews part was used to survey the information on family numbers, economic status of family, family disease history etc. The individual interviews part aimed to investigate the demographics variables of individuals (such as gender, age, nationality, occupation, education level, marital status) as well as the status of smoking, drinking, daily diet, history diagnosed, and knowledge of chronic diseases (such as hypertension, diabetes, hyperlipaemia, myocardial infarction and stroke).

The healthy lifestyle intervention questionnaires were designed to survey the implementation situation of healthy lifestyle intervention advocated by the WHO, such as dietary habit, salt intake, active smoking, passive smoking, alcohol drinking, physical exercise, sedentary behavior and sleep time etc.

Face to face interview was used to collect the data. Each individual was interviewed separately by one trained interviewer in a private place of the hospital or the individual’s home after signing the informed consent. The average time for each interview was about an hour.

### Physical and biochemical indicators examination

The tapeline, weighing-machine, sphygmomanometer and echoscope were used by the village health agencies clinical staffs to measure the physical indicators, such as height, waistline, weight, blood pressure and heart rate.

First the clinical laboratory doctors came from the county hospital asked whether the participant was taking oral hyperglycaemic agents, if not then the doctor drew the blood sample from each individual before the breakfast. The blood tubes samples were spun within an hour and rapidly transported to local county hospital clinical laboratory under the cold chain environment. The blood samples drawn from cubital vein were used to measure the biological indicators, such as fasting blood glucose (FBG), triglyceride (TG), total cholesterol (TC), high-density lipoprotein (HDL-C), and low-density lipoprotein (LDL-C).

### Diagnostic criteria

The diagnostic criteria of hypertension were as follows: if systolic pressure ≥ 160 mmHg or/and diastolic pressure ≥ 95 mmHg then diagnosed with hypertension; if 140 mmHg < systolic pressure < 160 mmHg or/and 90 mmHg < diastolic pressure < 95 mmHg then diagnosed with borderline hypertension.

The diagnostic criteria of diabetes were as follows: if fasting blood-glucose (FBG) ≥ 7.0 mmol/L then diagnosed with diabetes; if fasting blood-glucose (FBG) < 3.9 mmol/L then diagnosed with hypoglycemia.

The diagnostic criteria of hyperlipaemia are as follows: if total cholesterol (TC) > 5.72 mmol/L (220 mg/dL) or/and triglyceride (TG) > 1.70 mmol/L (155 mg/dL) then diagnosed with hyperlipaemia; if 5.2 mmol/L (200 mg/dL) < total cholesterol (TC) < 5.72 mmol/L (220 mg/dL) then diagnosed with borderline hyperlipemia.

### Ethics approval and consent for participate statement

This study was approved by the Ethics Committee of Weifang Medical University, and written informed consent was obtained from all participants.

### Statistical analysis

The mean and standard deviation ($\overline{x}\pm SD$) and frequency (%) were used to describe the distribution of quantitative and qualitative data respectively. The chi-square test was used to compare the difference of prevalence between male and female groups. All analyses were two tailed and statistical significant probability was determined by *P* ≤ 0.05. SPSS (Version 19.0) statistical analysis software was used to analyze data.

## Results

### Demographics description of the sample

There were 2480 eligible residents recruited in the current study and 2168 residents actually participated in. The participation rate was 87.42%. Demographic variables such as gender, age, occupation, education level, marital status and family annual income were shown in Table 1. The result indicated that gender variable was similar (male 46.6% VS female 53.4%). The age of interviewed residents mainly ranges from 41 to 80 years old. 76.6% of the samples were engaged in agriculture. As to the education level, 56.2% of the samples were under or equal primary school, which showed that the education level of the sample was relatively low in the current study. 88.2% of the samples were married or cohabitation. The mean of the family annual income was about 28,252 *yuan* (China’s per capita disposable income of the year was 11,886 *yuan*), which indicated that the income level of sample was lower than the average level of China.

**Table 1:** Distribution of the Demographics Variables

Demographic Variables	Frequency	Percent (%)
Gender		
Male (1)	1009	46.6
Female (2)	1158	53.4
Age (year)		
≤ 20 (1)	14	0.6
21–40 (2)	236	10.9
41–60 (3)	1004	46.3
61–80 (4)	779	35.9
≥ 81 (5)	135	6.2
Occupation		
Agriculture (1)	1659	76.6
Non- agriculture (2)	509	23.4
Education level		
No formal education (1)	597	27.5
Primary school (2)	623	28.7
Middle school (3)	915	42.2
Above middle school (4)	32	1.5
Marital Status		
Never married (1)	66	3.0
Married or cohabitation (2)	1912	88.2
Lose spouse, divorced or separation (3)	189	8.7
Family Annual Income (yuan)) ($\overline{x}\pm SD$)	28,252.18 ± 27,104.17^a^	

Note: ^a^indicated mean and standard deviation

### Prevalence of chronic disease

Table 2 showed the prevalence of common chronic disease for the total sample, male and female in rural China. It indicated that there were statistical differences on history diagnosed of hypertension, diabetes between male and female. The current prevalence of diabetes, hyperlipidemia between male and female were still statistical different.

**Table 2:** Prevalence of Common Chronic Disease in Rural China ^a^

**Chronic Disease**	**Variables**	**Total sample**	**Freq (%)**		***χ***^**2**^	***P***
			**Male**	**Female**		
**Hypertension**	History Diagnosed				6.008	**0.014**
	Yes (1)	517 (29.36)	213 (26.5)	304 (31.8)		
	No (2)	1244 (70.64)	592 (73.5)	652 (68.2)		
	Current Diagnosed				0.600	0.741
	Normal or below (1)	1052 (65.02)	470 (65.1)	582 (65.0)		
	Borderline (2)	162 (10.01)	68 (9.4)	94 (10.5)		
	Hypertension (3)	404 (24.97)	184 (25.5)	220 (24.6)		
**Diabetes**	History Diagnosed				15.457	**< 0.001**
	Yes (1)	107 (15.03)	28 (9.0)	79 (19.7)		
	No (2)	605 (84.97)	282 (91.0)	323 (80.3)		
	Current Diagnosed				7.811	**0.020**
	Hypoglycemia (1)	31 (1.87)	19 (2.6)	12 (1.3)		
	Normal (2)	1500 (90.53)	681 (91.4)	819 (89.8)		
	Diabetes (3)	126 (7.60)	45 (6.0)	81 (8.9)		
**Hyperlipidemia**	History Diagnosed				3.373	0.066
	Yes (1)	122 (5.68)	47 (4.7)	75 (6.5)		
	No (2)	2027 (94.32)	954 (95.3)	1073 (93.5)		
	Current Diagnosed				28.805	**< 0.001**
	Normal (1)	762 (46.49)	392 (53.1)	370 (41.1)		
	Borderline (2)	217 (13.24)	100 (13.6)	117 (13.0)		
	Hyperlipaemia (3)	660 (40.27)	246 (33.3)	414 (45.9)		
**Myocardial Infarction**	Suffered in Past one Year					
	Yes (1)	23 (1.06)	11 (1.1)	12 (1.0)	0.016	0.900
	No (2)	2139 (98.94)	994 (98.9)	1143 (99.0)		
**Stroke**	Suffered in Past one Year					
	Yes (1)	45 (2.09)	20 (2.0)	25 (2.2)	0.083	0.773
	No (2)	2111 (97.91)	984 (98.0)	1127 (97.8)		

Note: ^a^indicated exist missing data

The prevalence of history and current diagnosed hypertension were 29.36 and 24.97% in rural China. The prevalence of history diagnosed hypertension for female was higher than male (female 31.8% VS male 26.5%), but current prevalence of diagnosed hypertension was not statistically different between two groups (female 24.6% VS male 25.5%).

The prevalence of history and current diagnosed diabetes were 15.03 and 7.60% in rural China. And the prevalence of history and current diagnosed of diabetes for female were both higher than male (female 19.7% VS male 9.0%, female 8.9% VS male 6.0%).

The prevalence of history and current diagnosed hyperlipidemia were 5.68 and 40.27% in rural China. The prevalence of history diagnosed of hyperlipidemia for female and male were 6.5 and 4.7% respectively. The prevalence of current diagnosed of hyperlipidemia for female was higher than male (female 45.9% VS male 33.3%).

The one-year prevalence of myocardial infarction (MI) for total sample was 1.06%, for female was 1.0% and for male was 1.1%. The one-year prevalence of stroke for total sample was 2.09%, for female was 2.2% and for male was 2.0% respectively.

### Healthy lifestyle intervention related to chronic disease

Table 3 exhibited the major healthy lifestyle intervention variable related to chronic disease. There were significant differences on history smoking, current smoking, alcohol drinking, high physical work, and middle physical work between male and female.

**Table 3:** Healthy Lifestyle Intervention Variables Related to Chronic Disease ^a^

**Variables**	**Total sample**	**Freq (%)**		***χ***^**2**^	***P***
		**Male**	**Female**		
History Active Smoking				592.313	< 0.001
Smoking everyday (1)	501 (25.56)	443 (50.7)	58 (5.3)		
Smoking but not every day (2)	70 (3.57)	54 (6.2)	16 (1.5)		
No smoking (3)	1389 (70.87)	376 (43.1)	1013 (93.2)		
Current Active Smoking				530.170	< 0.001
Smoking everyday (1)	512 (23.65)	457 (45.3)	55 (4.8)		
Smoking but not every day (2)	44 (2.03)	35 (3.5)	9 (0.8)		
No smoking (3)	1609 (74.32)	516 (51.2)	1093 (94.5)		
Passive Smoking				1.170	0.279
Yes (1)	879 (42.65)	413 (43.9)	466 (41.6)		
No (2)	1182 (57.35)	527 (56.1)	655 (58.4)		
Alcohol Drinking				665.228	< 0.001
Drinking within a month (1)	600 (27.86)	522 (51.9)	78 (6.8)		
Drinking but a month ago (2)	96 (4.46)	82 (8.2)	14 (1.2)		
No Drinking within a year (3)	1458 (67.69)	401 (39.9)	1057 (92.0)		
Control Salt Daily Intakes Action				0.668	0.414
Yes (1)	1014 (47.01)	463 (46.1)	551 (47.8)		
No (2)	1143 (52.99)	542 (53.9)	601 (52.2)		
High Intensity Physical Work				4.715	0.030
Yes (1)	921 (42.70)	454 (45.2)	467 (40.5)		
No (2)	1236 (57.30)	551 (54.8)	685 (59.5)		
Mild Intensity Physical Work				87.244	< 0.001
Yes (1)	1573 (72.82)	637 (63.3)	936 (81.2)		
No (2)	587 (27.18)	370 (36.7)	217 (18.8)		
High Intensity Physical Exercise				0.293	0.588
Yes (1)	23 (1.07)	12 (1.2)	11 (1.0)		
No (2)	2122 (98.97)	987 (98.8)	1135 (99.0)		
Mild Intensity Physical Exercise				0.075	0.784
Yes (1)	169 (7.89)	77 (7.7)	92 (8.0)		
No (2)	1974 (92.11)	921 (92.3)	1053 (92.0)		
Sedentary Behavior Time (Hours/per day)	3.90 ± 2.50	3.90 ± 2.53	3.89 ± 2.48	0.114	0.910
Sleep Time (Hours/per day)	7.72 ± 2.03	7.77 ± 1.41	7.68 ± 1.40	1.416	0.157

Note: ^a^indicated exist missing data

#### Active smoking and passive smoking

The history active smoking rate of total sample was 29.13% and current active smoking rate was 25.68%. The male was higher than female both on history active smoking rate (male 56.9% VS female 6.8%) and current active smoking rate (male 48.8% VS female 5.6%). The passive smoking rate of total sample was 42.65%, and male 43.9%, female 41.6%.

#### Alcohol drinking and salt intake

There was 27.86% drunk alcohol within a month and 4.46% a month ago of total sample. 51.9% male and 6.8% female drunk alcohol within a month, which was statistically different between two groups (*P* < 0.001). 47.01% participated in controlling salt daily intakes action (≤6 g/day) in total sample. Only 46.1% male and 47.8% female participated in the control salt daily intakes action (≤6 g/day).

#### Physical work and physical exercise

The result indicated that 42.70 and 72.82% persons engaged in high and mild intensity physical work of the total sample. The male engaged more high intensity physical work than female (male 45.2% VS female 40.5%), but the female engaged more mild intensity physical work than male (male 63.3% VS female 81.2%). When it comes to the physical exercise, only 1.07 and 7.89% population attended high and mild intensity physical exercise of total sample. Only 1.2% male and 1.0% female took high intensity physical exercise and 7.7% male and 8.0% female took mild intensity physical exercise. There were no significant differences on high and mild intensity physical exercise between male and female.

#### Sedentary behavior and sleep time

The current study indicated that the mean and the SD of sedentary behavior time and sleep time were 3.90 ± 2.50 and 7.72 ± 2.03 of the total sample. There were no statistically significant differences both on sedentary behavior time and sleep time between male and female.

## Discussion

Cross-sectional survey was carried out to research the prevalence of chronic diseases and the implementation of healthy lifestyle intervention in current study. Most of the participants were peasants and the middle-aged and elderly, which was the real situation in current rural China, because most of the Youngers flock to work in urban areas with the rapid development of urbanization in China. The education level of retained rural inhabitants was relatively low and the family annual income was not high enough. The prevalence of chronic diseases and awareness of healthy lifestyle intervention were related to education and income level. Relatively low education and poor residents always have inadequate knowledge on dangers of chronic disease and the awareness of healthy lifestyle intervention.

### The prevalence of chronic diseases

The current prevalence of hypertension of total sample, female, male were 24.97, 24.6, and 25.5%. The hypertension prevalence of total sample was lower than the rural area of Northwest China (30.5%) [22] and the 2007–2008 China national diabetes and metabolic disorders study (26.6%) [23]. The female hypertension prevalence was similar to the previous study (24.0%) and male hypertension prevalence was lower than the previous study (29.2%) [23].

The current prevalence of diagnosed diabetes for total sample, female, male were 7.60, 8.9, 6.0% respectively, which was lower than the Chinese disease surveillance 2010 (11.6%) [9]. The different results may be derived from different survey samples. The samples of current study came from rural area and the subjects took up physical labor every day, which may be the major reason for lower prevalence of diabetes. Another reason maybe the effect of China’s basic public health service project implemented in recent years. Compare the history diagnosed prevalence (15.03%) with the current diagnosed prevalence (7.60%), the declining trend was obvious. It maybe benefited from the enhancement of diabetes preventive awareness in recent years, in rural areas of China.

Be of great concern, the current prevalence of hyperlipidemia (total sample 40.27%, female 6.5%, male 4.7%) increased greatly than history prevalence (total sample 5.68%, female 45.9%, male 33.3%). There were some possible reasons explaining the obviously rising tendency.

First, with the rapid development of economy in rural areas of China, more and more nutritious foods such as animal fat and high-protein eggs are easily available in daily life. Second, the syndrome of hyperlipidemia is not obvious and serious enough, so the measurement and supervision of blood lipid is ignored. Third, because the low income and the paid blood lipid monitoring, blood lipid examination is hard to be carried out and popularized in rural China. So, most of the inhabitants had never measured their blood lipid before, and the vast majority of these were newly detected. The rapid increase of hyperlipidemia prevalence maybe profited from the laboratory diagnostic technique and awareness promotion of hyperlipidemia. The newly discovered hyperlipidemia cases may be only the tip of iceberg in rural China. The current research suggests that low fat diet intervention should be strengthened and the epidemiological screening for hyperlipidemia is necessary and urgent.

### Healthy lifestyle interventions related to chronic diseases

The effective implementation of healthy lifestyle intervention should be paid more attention to in the future. People who had never measured their blood pressure and blood glucose account for about 20.0% and above 70.0% respectively. Those phenomena manifest that the healthy consciousness is still defective in rural areas of Shandong province, China. Compared with the history smoking, it was easy to find that the active smoking reduced but slightly. Meanwhile, there was 48.8% male active smoking and passive smoking rate of the total sample was 42.65%. The current active smoking prevalence in the sample was higher than the previous literature (26.2%) [17]. So the active smoking and passive smoking were still serious in rural china. Above half of male drunk alcohol within a month and less half of inhabitant took part in control salt daily intakes action. The previous study had indicated that excessive alcohol use was related to the premature deaths involving chronic diseases [24, 25], so excessive alcohol use was the risk factor of chronic diseases and harmful to the health. As is well known that high salt intake results in hypertension and stroke, but there was still over 50% people hesitate to reduce salt intake.

Another phenomenon should be paid close attention to was physical exercises. Only 1.07 and 7.89% attended high and mild intensity physical exercises. In spite of rural residents engage in physical work every day, they spent little time on physical exercises to prevent chronic diseases. Regular and moderate physical exercises would decrease the risks of chronic disease, such as cardiovascular disease, hypertension, diabetes, stroke and cancer etc. [26–30]. But, the implication of physical exercise was not optimal in rural China.

### Countermeasures and proposals

The smoking, intemperance, high salt intake and lack of exercise have been validated as the risk factors for chronic diseases, but the healthy lifestyles interventions advocated by the government, bureau and media to prevent disease have not got desired effective results in rural daily life. It suggests that the basic healthy lifestyle intervention including low fat diet, control cigarettes, quit alcohol, low salt and appropriate amount of exercises should be strengthened and the challenge of chronic diseases prevention is still serious in rural China.

As to the implications, this study would provide theoretical foundation for the near or long-term public health projects basing on China’s interests in the future. It is useful to prevent and control chronic diseases in rural areas in China. The government, medical institutes and individuals should adopt effective measurement to reduce the epidemic situation of chronic diseases firmly. Because about 80% of the population is rural residents in China, related health policy should be promulgated leading the health resources such as health funding, advanced technical and professional village doctors flung to rural areas. More perfect and multiple channels financing medical care insurance system should be carried out by the national government to reduce the economic burden of chronic disease in rural areas, China [31]. The epidemiological screen for chronic disease, especially for hyperlipidemia, should be strengthened in rural China. Various aspects of society, such as media, sanitary bureau, health care institution, hospital, medical insurance section, local health village doctors should enhance publicity of healthy lifestyles interventions in order to enhance the healthy awareness of the population. The projects of health education and health promotion still need to be strengthened, four bases of health (rational diet, regular exercises, no smoking and limited amount of liquor, and psychological balance.) initiated by WHO should be propagandized extensively. Regular physical examination, standardized early treatment and long-term health care [15] for high risk population of chronic diseases all still need to be strengthened urgently.

### Limitations

Forthcoming studies based on larger scale of sample on different age population needed to be carried out in the future. The inherent inaccuracy of self-report survey may results in research bias. There was no compared area, so standardized prevalence was not calculated in the current study.

## Conclusions

The prevalence of chronic diseases was still high, especially hyperlipidemia, and the challenges to prevent chronic diseases was still severe. The implementation of healthy lifestyle intervention for chronic diseases was not optimal in rural areas in China. Challenges to prevent chronic diseases and the implementation of healthy lifestyle intervention were still severe, so medical institutes, government and individuals should put into effective strategy to reduce the epidemic of chronic diseases and health promotion project should be effectively strengthened. Comprehensive prevention strategies and health promotion project should be effectively strengthened in order to prevent the epidemic of common chronic diseases. The current study has significances for improving public health in China and to guide near or long-term projects of public health in the future.

## Data Availability

The datasets generated and analyzed of the current study are not publicly available due to the funding regulations but are available from the corresponding author on reasonable request.

## Abbreviations

CDC: Center for Disease Control and Prevention
TG: Ttriglyceride
TC: Total cholesterol
HDL-C: High-density lipoprotein
LDL-C: Low-density lipoprotein
FBG: Fasting blood-glucose
